# Improved drag coefficient and settling velocity for carbonate sands

**DOI:** 10.1038/s41598-020-65741-3

**Published:** 2020-06-11

**Authors:** Amin Riazi, Ana Vila-Concejo, Tristan Salles, Umut Türker

**Affiliations:** 1 Civil Engineering Department, Cyprus International University, Lefkoşa, 99258 North Cyprus Turkey; 2 Geocoastal Research Group, School of Geosciences, The University of Sydney, Sydney, 2006 Australia; 3 Civil Engineering Department, Eastern Mediterranean University, Gazimağusa, 99628 North Cyprus Turkey

**Keywords:** Geomorphology, Civil engineering

## Abstract

Sediment transport calculations are used globally in the numerical models that coastal managers, scientists and engineers use to assess and forecast coastal change. Most of the existing sediment transport equations were defined based on experimental results using siliciclastic sands. Yet these equations are applied to all types of sand, including carbonate sands that have different characteristics and therefore, settling behaviour. A rigorous management of the transport of carbonate sand is essential for the present and future management of sedimentary features in coral reefs such as sandy beaches or reef islands. Here we present a new approach to estimating the drag coefficient of carbonate sands that considers both friction and pressure. Based on our new method, the calculated drag coefficients explain the great variability in settling velocities of carbonate sand observed in nature (from 0.025 m/s to 0.364 m/s in our database). Using our formula, we demonstrate that even small differences in the settling velocity obtained with the new drag coefficient can lead to substantial changes in sediment transport and call for an update of numerical models.

## Introduction

Coral reefs are three-dimensional structures that comprise fine veneers of living coral colonies and other organisms overlying vast sequences of dead coral, as such they are mainly composed of sedimentary deposits – carbonate sand and rubble. These deposits are highly mobile, and their changing morphologies can influence the characteristics of the living regions of coral reefs and other biota. An example of these sedimentary deposits are the vulnerable coral-reef islands whose fate directly related to the effects of climate change (including sea-level rise, ocean warming and acidification and changes in wave climate), has been the subject of recent investigation and debate^1–4^. Much of that debate is subject to the correct understanding of how carbonate sediment is transported. Sediment properties such as size, shape and density are ultimately responsible for the thresholds at which sediment becomes entrained and transported through different mechanisms. Understanding how, why and when sediments move is crucial to managing and predicting the effects of climate change, and, allows us to prepare mitigation/adaptation strategies. Most sediment transport studies have focused on siliciclastic (quartz) sands with many studies dedicated to analysing settling velocity and drag coefficients^5–7^. In fact, the influence of sediment variability has been mostly ignored even though the majority of the world’s coast have heterogeneous sediments^8^. Carbonate sediments are mostly derived from the physical and chemical breakdown of biogenic structures (e.g., coral) and the death of organisms (e.g., foraminifera, *Halimeda*) and, therefore, they have highly irregular shapes and densities^9^. Researchers have acknowledged that the different shape and density of carbonate sands have important implications for the hydraulic properties that control sediment transport^9–16^; for example Smith and Cheung^15^ demonstrated that irregular particles were more easily entrained under rough turbulent flow than regular particles with equivalent diameter. Here we present a novel study on the hydraulic properties of carbonate sand that shows, while settling velocity can be improved by incorporating a particle shape factor, improvements are much greater when using a new formulation for drag coefficient for carbonate sands that includes both a frictional and a pressure drag coefficient. This new drag coefficient equation is optimised using genetic algorithms^17^. We use the characteristics of carbonate particles from Heron Island (Great Barrier Reef) and from Oahu, Hawaii which have been extensively analysed in the literature^14^. Finally, we quantify the implications that using our improved settling velocity equations have for estimating sediment transport modes (wash, suspended, and bed load) using the Rouse number for 5 years of wave-induced bed shear stress on the North Shore beaches of Oahu, Hawai’i.

## Carbonate Particles are not Spheres, they are best Represented by Ellipsoids

**Figure 1:**
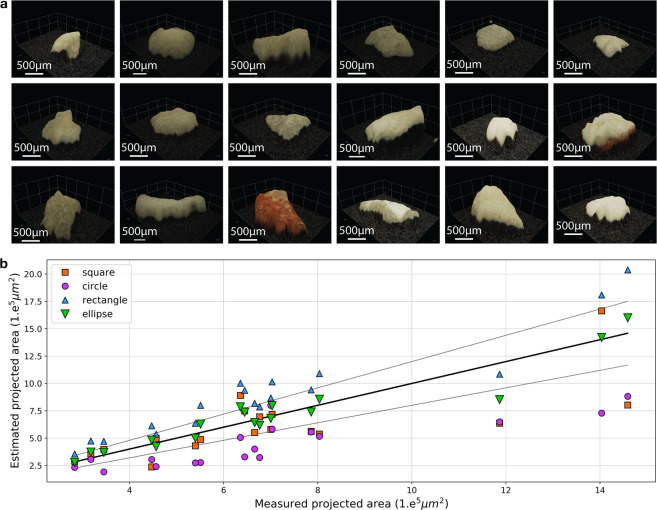
The shape of carbonate sands. (**a**) 3D scans of the 18 Heron particles using a Hirox RH2000 microscope equipped with MXB2016Z. (**b**) Estimation of the projected area of the particles by considering the particles as different geometric shapes (squares, circles, rectangles, or ellipses).

Due to their organic origin, carbonate sands exist on a variety of shapes. Settling velocity studies have either considered particles to be spheres^5^, or have incorporated the shape of the particles in complex empirical drag coefficient formulae^18,19^. We analysed the shape of 18 random particles obtained from the intertidal (active) region of a beach in Heron Island (Southern Great Barrier Reef) and found that an ellipse was the best representation of their projected area with a mean relative error of 10.04% (Fig. 1). We found that the traditional circle assumption typically yielded an underestimation of the projected area (Fig. 1b) with a mean relative error of 35.30% that leads to the overestimation of the drag coefficient. The comparison of the shape of our Heron particles with those particles from Smith and Cheung’s^14^ dataset (fine to very coarse calcareous grains obtained from 13 beaches on Oahu, Hawaii) showed that they were extremely similar [Sup Table S1] and, therefore, we assume that those would be best represented by ellipsoids instead of spheres.

### Settling velocity equation for carbonate sands

The settling velocity of sediment particles (*ω*) is derived by equating the effective weight force to the drag force (Eq. 1).

1 $${\omega }^{2}=2\frac{(S-1)g}{{C}_{D}}\frac{V}{{A}_{p}}$$

where, *V* is the volume, *A*_*p*_ is the projected area, *S* is the specific gravity of the sediment particle, *g* is the gravitational acceleration, and, *C*_*D*_ is the drag coefficient. Most researchers have used Eq. 1 assuming sediment particles to have a spherical shape, therefore, the term $V/{A}_{p}$ equals *2/3 d*_*n*_, where *d*_*n*_ is the particle nominal diameter. The most commonly accepted empirical relationship between the drag coefficient and the particle Reynolds number $(R{e}_{p})$ was proposed by Cheng^5^ for siliciclastic sands (Eq. 2).

2 $${C}_{D}={\left[{\left(\frac{A}{R{e}_{p}}\right)}^{\frac{1}{m}}+{B}^{\frac{1}{m}}\right]}^{m}$$

where, *A*, *B*, and *m* are constants and the particle Reynolds number is given by $R{e}_{p}=\omega {d}_{n}{\nu }^{-1}\,,$ with *v* the kinematic viscosity of ambient water. Wu and Wang^19^ combined Eqs. 1 and 2 and obtained Eq. 3 for siliciclastic sands.

3 $$\omega =\frac{A\nu }{B{d}_{n}}{\left[{\left(\frac{1}{4}+{\left(\frac{4B}{3{A}^{2}}{D}_{\ast }^{3}\right)}^{\frac{1}{m}}\right)}^{\frac{1}{2}}-0.5\right]}^{m}$$

where, D_*_ is the non-dimensional particle size as described by Wadell^20^ and Soulsby^21^ (Eq. 4):

4 $${D}_{\ast }={d}_{n}{\left[\frac{(S-1)g}{{\nu }^{2}}\right]}^{\frac{1}{3}}$$

where, *d*_*n*_ for ellipsoids is calculated as ${d}_{n}={({d}_{l}{d}_{i}{d}_{s})}^{\frac{1}{3}}$, considering *d*_*l*_, *d*_*i*_, and *d*_*s*_ are the diameters in the longest, intermediate, and the shortest mutually perpendicular axes, respectively.

Wu and Wang^19^ introduced the effect of the particles shape in Eq. 2 by incorporating the Corey Shape factor *S*_*f*_ (Eq. 5)^22^ to develop expressions for coefficients *A*, *B*, and *m* such as $A=53.5{e}^{-0.65{S}_{f}};B=5.65{e}^{-2.5{S}_{f}};m=0.7+0.9{S}_{f}$.

5 $${S}_{f}=\frac{{d}_{s}}{\sqrt{{d}_{l}\times {d}_{i}}}$$

Recently, Riazi and Türker^23^ undertook a comprehensive study on the shape of siliciclastic sands where they found that it was more accurate to assume the particles to be ellipsoidal instead of spherical. They developed Eq. 6 introducing the Corey shape factor directly in the settling velocity equation and improving the ratio of volume to projected area for natural siliciclastic particles:

6 $${\omega }^{2}=\frac{4}{3}\frac{(S-1)g}{{C}_{D}}{{S}_{f}}^{\frac{2}{3}}{d}_{n}$$

Our results for carbonate sands indicate that these particles are also best represented by ellipsoids; however, as their shapes are more heterogeneous than siliciclastic sands, we found that adding an empirically derived constant *α* with value within (0,1], improves the volume over projected area estimations. Therefore, the *V*/*A*_*p*_ from Eq. 1 can be estimated with a high degree of accuracy by Eq. 7, using both our constant *α* and the Corey shape factor as demonstrated by Riazi and Türker^23^:

7 $$\frac{V}{{A}_{p}}=\alpha \frac{4}{6}{{S}_{f}}^{\frac{2}{3}}{d}_{n}$$

Optimisation of Eq. 7 using our dataset yielded 0.55 as the best value for *α*, resulting in the lowest error for *V*/*A*_*p*_. For example, just for our 18 samples from Heron Island (Fig. 1) this error reduced from 60.45% to 15.36%. By inserting Eq. 7 in to Eq. 1 we derived a new equation for settling velocity for carbonate sands (Eq. 8) that considers both the shape of the particles, and *α* with our optimal value of 0.55.

8 $${\omega }^{2}=\frac{11}{15}\frac{(S-1)g}{{C}_{D}}{{S}_{f}}^{\frac{2}{3}}{d}_{n}$$

Beside the drag coefficient, *C*_*D*_, the main difference between Eqs. 6 and 8, is *V/A*_*P*_ with respect to particle shape. The correct ratio for *V/A*_*P*_ is important as it affects the value of *C*_*D*_ significantly. For example, using Eq. 6 instead of Eq. 8, for carbonate sands will force the empirical equations for *C*_*D*_ to overestimate the drag coefficient to have more accurate settling velocity estimations.

### Effect of drag coefficient in settling velocity

*C*_*D*_ is generally considered a function of the particle Reynolds number (similar to Eq. 2) and therefore, it needs to be calculated in an iterative process^24^. However, current approaches estimate *C*_*D*_ directly (without any iterative processes), thus increasing the uncertainty of the calculations. Guo^7^ demonstrated that *C*_*D*_ for a given particle can be independent of the particle’s settling velocity. Recently, Riazi and Türker^23^ considered the drag coefficient (*C*_*D*_) as a dimensionless quantity that describes the resistance of a particle in a fluid environment^25^, and proposed a new equation for *C*_*D*_ that is calculated without directly using the Reynolds number or the settling velocity (Eq. 9):

9 $${C}_{D}={\left(\frac{{a}_{1}\times \nu }{{d}_{n}^{1.5}\times {g}^{0.5}}+{a}_{2}\right)}^{{a}_{3}}$$

where, *a*_1_, *a*_2_, and *a*_3_ are shape dependent constants calibrated for silica sands^23^ and calculated as ${a}_{1}=12.617{S}_{f}+17.61$; ${a}_{2}=\mathrm{-0.151}{S}_{f}^{14.167}+1.503{S}_{f}^{-0.147}-0.771$; ${a}_{3}=1.434$

**Figure 2:**
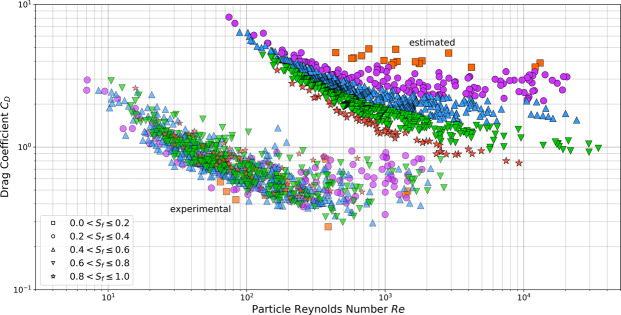
Drag coefficients for carbonate sediments as a function of the particle Reynolds numbers based on the Smith and Cheung’s^14^ dataset. Equation 2 has been used to estimate the values and Eq. 8 to obtain the experimental values. The shape of the particles is given using the Corey shape factor (*S*_*f*_).

Equation 2 indicates that as the particle Reynolds number increases, *A*/*Re*_*p*_ will approach zero. Therefore, for particles with large $R{e}_{p}$, the magnitude of *C*_*D*_ independent of sediment type (silica or carbonate) will depend solely on *B* showing an inherent asymptotic behaviour. However, for carbonate sands, based on the results from Smith and Cheung^14^, the *C*_*D*_ does not show a clear asymptotic behaviour when plotting the experimental *C*_*D*_ as a function of $R{e}_{p}$ (Fig. 2, experimental). Furthermore, the broad scatter of the points in Fig. 2 (experimental) indicates that Corey shape factor is not sufficient to describe the carbonate sands shape and thus, it does not affect the *C*_*D*_. We can therefore, remove particle shape (represented as Corey shape factor) from the calculation of *C*_*D*_ and express it as a function of particle nominal diameter, ambient fluid kinematic viscosity, and gravitational acceleration (Eq. 10).

10 $${C}_{D}=f({d}_{n},\nu ,\,g)$$

In Fig. 2 the drawback of using silica sands drag coefficient equation to estimate the drag coefficients of carbonate sands is notable. The silica sand approaches (estimated) force the results to obey the same pattern, however, the experimental results show that carbonate sands have completely different behaviour. Moreover, as it can be seen in the experimental section, for low $R{e}_{p}$ there is a linear decrease in both the estimated and the experimental ${C}_{D}$ as the $R{e}_{p}$ increases. However, for high values of $R{e}_{p}\,$ $(R{e}_{p} > 500)$, the estimated ${C}_{D}$ seems to increase arbitrarily as $R{e}_{p}$ increases. We can explain the slow settling of particles with large *C*_*D*_ and low $R{e}_{p}$, with the viscous drag of the laminar flow around each particle. The rapid settling of particles with low ${C}_{D}$ is predominantly resisted by the turbulent drag of the wake behind each particle^6^. Hence, depending on the particle’s Reynolds number, the drag force acting on a particle parallel to the direction of motion should take into account two components: (1) the frictional drag force depending on frictional drag coefficient; and, (2) the pressure drag force depending on pressure drag coefficient^26^. Therefore, Eq. 10 was expanded to cover both low and high Reynolds number and was made dimensionless with the help of Buckingham Π theorem, Eq. 11:

11 $${C}_{D}={C}_{D1}+{C}_{D2}={\left(\frac{{a}_{3}\times \nu }{{d}_{n}^{1.5}\times {g}^{0.5}}+{a}_{4}\right)}^{{a}_{5}}+{\left(\frac{{a}_{6}\times \nu }{{d}_{n}^{1.5}\times {g}^{0.5}}+{a}_{7}\right)}^{{a}_{8}}$$

**Figure 3:**
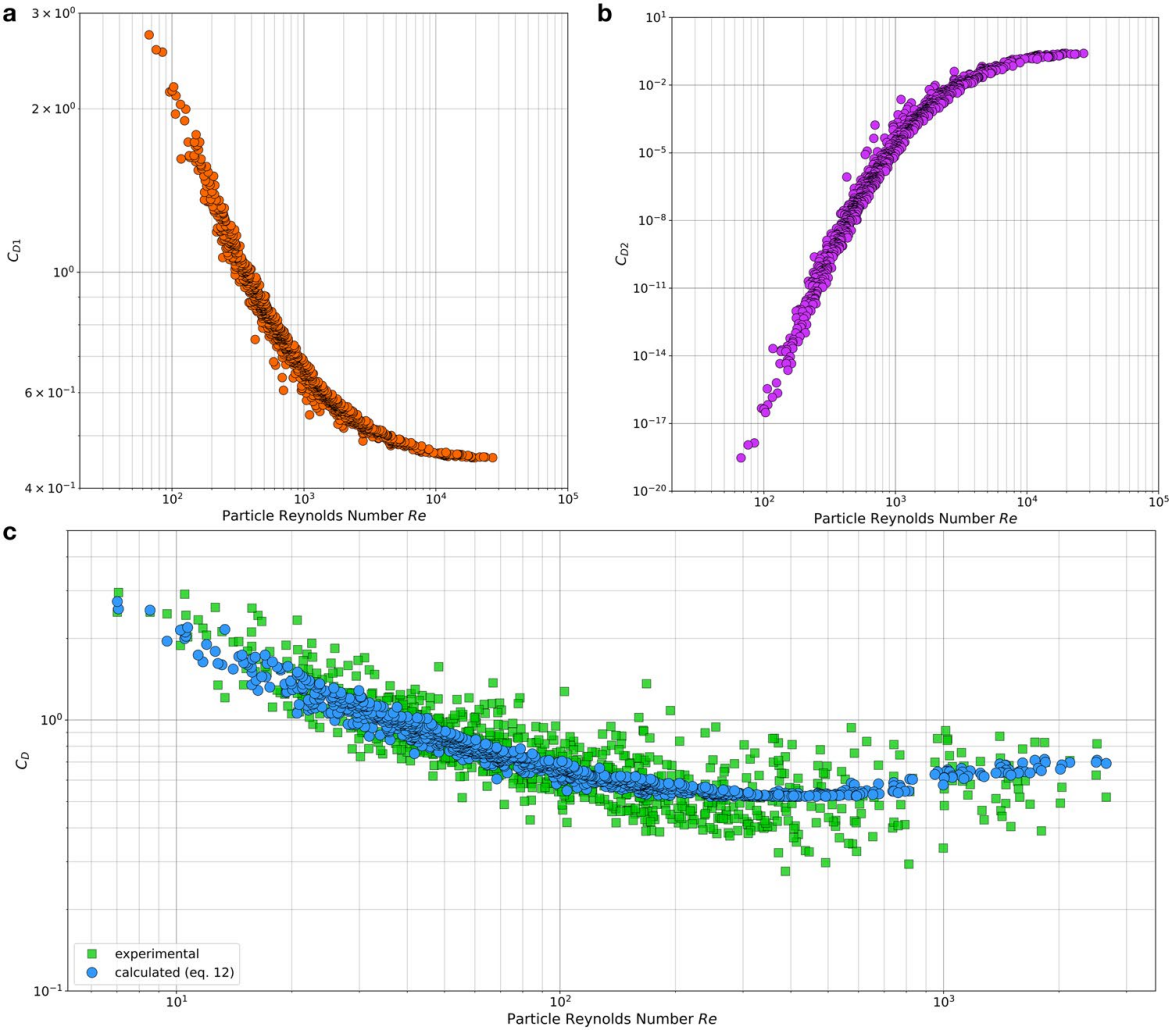
Drag coefficient calculations for carbonate particles. (**a**) Frictional drag coefficient (*C*_*D1*_) decreases as particle Reynolds number increases up to a constant asymptotic. (**b**) Pressure drag coefficient (*C*_*D2*_) increases as the particle Reynolds number increases. (**c**) Total *C*_*D*_ calculated as the summation of (**a**,**b**) following Eq. 12.

Equation 11 shows the drag coefficient (*C*_*D*_) as the summation of the two different drag behaviours described above. We then applied genetic algorithms as described in Riazi and Türker^17^, to optimise the value of the constants, thus obtaining Eq. 12. It is important to note that the power of the drag coefficients, *C*_*D1*_ and *C*_*D2*_, are significantly different. As it is shown in Fig. 3, the first part of the equation (*C*_*D1*_), representing the frictional drag coefficient, is dominant for low $R{e}_{p}$, while the second part (*C*_*D2*_), representing the pressure drag coefficient, is dominant for high $R{e}_{p}$. The settling velocity of carbonate sands is then obtained by inserting Eq. 12 into Eq. 8. The new *C*_*D*_ obtained with Eq. 12 yields the asymptotic behaviour expected when plotting *C*_*D*_ as a function of the particle Reynolds number (Fig. 3c).

12 $${C}_{D}=\,{C}_{D1}+{C}_{D2}={\left(\frac{9.50\times \nu }{{d}_{n}^{1.5}\times {g}^{0.5}}+0.76\right)}^{2.92}+{\left(\frac{20.47\times \nu }{{d}_{n}^{1.5}\times {g}^{0.5}}+1.02\right)}^{-48.15}$$

**Figure 4:**
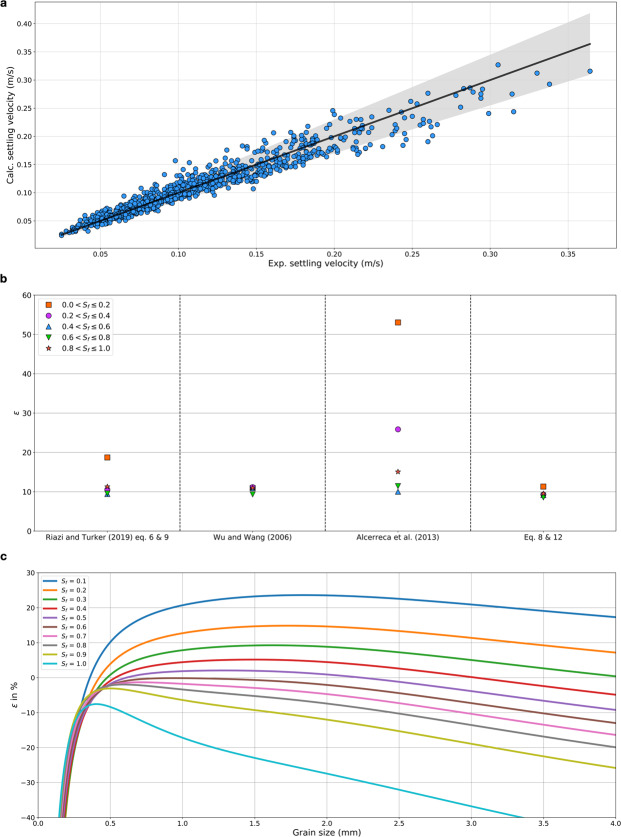
(**a**) Carbonate sands settling velocity calculated by Eq. 8; the shaded area represents ±15% error. (**b**) Accuracy of the different equations estimating settling velocity of carbonate sands over 938 samples from Smith and Cheung’s^14^ dataset. (**c**) Relative error calculated for a fixed specific gravity with different grain sizes and shape factors using Eq. 13 showing difference between the settling velocities estimated through carbonate and silica approaches. Negative values indicate that the silica approach estimates higher value of settling velocity than the carbonate approach.

We compared the measured settling velocity with the calculated settling velocity obtained using first Eq. 12 and then Eq. 8 (Fig. 4). The calculated settling velocities were more accurate for the slow settling particles (low Reynold numbers, i.e. Re < 500 yielded 8.97% error) than for the fast ones (high Reynold numbers, i.e. Re > 500 yielded 9.75% error), but more than 80% of the particles fell within the 15% error from the experimental data (Fig. 4a). We assessed the accuracy of our proposed equations (Eqs. 12 and 8) to compare the results for drag coefficient and settling velocity and obtained errors below 10% for all Corey shape factors except for those particles with very low *S*_*f*_ for which we had only 17 particles (Fig. 4b and Sup Table S2).

For particles with the same shape, diameter, and specific gravity, the settling velocity obtained using the equation of siliciclastic sands (Eq. 6) yields larger settling velocities than using our equation for carbonate sands (Eq. 8).We then evaluated the relative errors (ε) for the different grain sizes and *S*_*f*_ (Eq. 13 and Fig. 4c). It is important to note that the size and shape of our particles are distributed such that 92% had sizes > 0.25 mm and ≤3 mm, and Sf > 0.2 and ≤0.8 (Fig. S1). Our results (Fig. 4c) show that ε was maximum for the smallest particles (<0.4 mm), regardless of *S*_*f*_. Within the sizes interval of (0.25, 3], the shape factors for which ε < 5% were 0.4 and 0.5; for *S*_*f*_ = 0.6 the ε of particles larger than 2.75 mm indicates an overestimation of the settling velocity by the silica approach. The ε is maximum for fine sediments corresponding to fine sand and below in the Wentworth grain size scale; and, for coarse sediments corresponding to very fine gravel and above in the same scale. For example, for a *S*_*f*_=0.8, the ε is less than 5% only for particles with sizes between 0.3 and 1.5 mm, with overestimation of settling velocity for the finest grains and underestimation for the coarsest grains (Fig. 4c).

13 $${\rm{\varepsilon }}=\frac{{\omega }_{calcareous}-{\omega }_{silica}}{{\omega }_{calcareous}}\times 100$$

### Implications for sediment transport

In practical applications to bioclastic environments, settling velocities are used to describe multiple reef zones and to predict sediment transport mode. These applications are critical to understand and predict sediment transport pathways through these systems^9^ but also to evaluate the damage to corals exposed to sedimentation^27,28^. In a world where sand and gravel are being extracted faster that they can be replaced^29^, it is crucial that we refine existing sediment transport calculations to minimise waste of sand.

To assess our improved settling velocity formulation and quantify its implication for sediment transport, we used North Shore Oahu (Hawai’i) as our study region (Figs. S3–1), wave data for a period spanning from 2011 to 2016 from the Pacific Islands Ocean Observing System (Figs. S3–2) (http://www.pacioos.org/) and grain size information from Hampton^30^. The mean grain size for the carbonate sands in the study area is 0.43 mm, with minimum size of 0.13 mm and maximum size of 1.07 mm. We obtained settling velocities using equations derived for siliciclastic sand (Eqs. 9 and 6) and with our equations (Eqs. 12 and 8) derived for carbonate sands. It is important to note that both the mean and the maximum grain size correspond to the range for which we found minimum ε (<5%) for most *S*_*f*_ (Fig. 4c) when comparing the settling velocities obtained with the siliciclastic equations *vs* those obtained with our carbonate equations; simultaneously, the minimum size corresponds to large ε in which the settling velocity is overestimated by the silica approach (Fig. 4c). We then used the same forcing to derive the different modes of transport (bed load, suspended load, and wash load) using the Rouse number^31^ and compared the results obtained when using the settling velocity derived with the siliciclastic equations with those obtained using the settling velocity derived from our carbonate equations.

The Oahu shelf sands are mainly carbonate with only a small percentage of terrigenous content^32–35^. These carbonate sands accumulate in relatively thin patches, fields, and linear deposits perched on the shallow shelf^36^. Above the 20 m depth, most sediment on the reef is produced by reef builders, reef dwellers, and reef bioeroders, making this zone the primary source of nearshore sands^37^. In this study, it is worth noting that we only evaluate the transport of loose sands and therefore do not estimate the impact of reef rugosity on frictional dissipation in the spectral wave model^38–40^ neither the effect of hard coral disintegration in loose particles.

When using the carbonate equations, the Rouse number showed that most of the transport occurred by bed load for the mean and maximum grain sizes considered (Figs. S3–3). For the minimum grain size diameter (0.13 mm), the estimated Rouse number mainly exhibited 2 modes of transport: wash load and bedload (Figs. S3–3). These results fit well with the prevailing major swell directions in the region (i.e. North Pacific swell during winter and Southern swell during summer).

**Figure 5:**
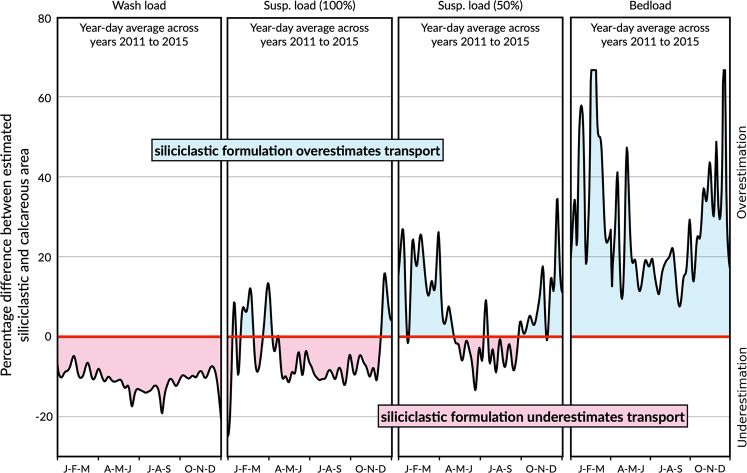
Comparisons of estimated modes of transport between the two settling velocity formulations for the mean grain size diameter (0.43 mm) based on the formulations of settling velocity for siliciclastic and carbonate sands ranging from January 2011 to January 2016 (details for each individual formulation are plotted in Figs. S3–3). Red centre line corresponds to the case where both formulations are equivalent. Divergence from this centre line shows overestimation of the siliciclastic formulation in respect to the carbonate one (blue shaded area) or underestimation (red shaded area).

Comparisons of estimated modes of transport for the two settling velocity formulations (Fig. 5) showed that the Rouse number predicted using the siliciclastic equations typically underestimates both the wash and suspended load modes and over predicts the percentage of bedload transport. It should be noted that the ε for a grain size of 0.43 mm are small (<5%) for 0.2 < Sf < 0.9 (Fig. 4c). Therefore, even when considering grain sizes with small ε, the implications for sediment transport estimates can be large. For example, a numerical model using the siliciclastic equations for the sand at Oahu could overestimate bed load by ~20%, while underestimating wash and suspended load by ~10% (Fig. 5).

## Methods

### Genetic algorithm optimization

Among different methods that can be used to optimize Eq. 11, herein, genetic algorithm as described in Riazi and Türker^17^ were used. In the proposed method by Riazi and Türker^17^ the search space is divided to smaller search zones. The number of search zones are directly related to the number of variables that should be optimized. As in Eq. 11 there are 6 unknowns, 2^6^ search zones were required. To increase the speed of optimization process, in each search zone two chromosomes were considered. After 1000 iterations, the optimized solution was obtained.

### Sediment transport mode analysis

#### Sediment settling velocities

Information on grain size distribution for the region has been derived from both vibracore metadata provided by USGS reef-front carbonate sediment deposits dataset^30^ and beach samples from the survey lines^30^. The grain size dataset was then converted from Θ-scale to millimetre. Given the sparse distribution of the dataset^32^ and to account for the full extent of carbonate sands grain sizes, we calculate our Rouse number analysis for three types of nominal diameter (geometric mean, minimum and maximum) set to 0.43, 0.13 and 1.07 mm, respectively. The Corey shape factor (*S*_*f*_) and the sediment density (ρ_s_), were considered fixed and equal to 0.556 and 2600 kg/m^3^, respectively following average values in Smith and Cheung’s^14^ dataset.

The settling velocity for carbonate sands is calculated for each grain size diameter using the drag coefficient (*C*_*D*_) and settling velocity (*ω*) proposed in Eqs. 12 and 8, respectively. Using similar values for *S*_*f*_ and ρ_s_, the settling velocity for siliciclastic sands is derived from the Eqs. 9 and 6 following Riazi and Türker^23^.

The settling velocity is then used to estimate entrainment and transport mode (bedload, suspended load, or wash load) in the region, using the Rouse number *P* = *w*_*s*_/κu_*_^32^ where, $\kappa$ is the von Karman constant (0.4) and u_*_ is the shear velocity estimated from the wave-induced bed shear stress τ_w_.

#### Wave-induced bottom shear stress

The wave dataset obtained from PacIOOS is solved with a spectral model SWAN^41^ and consists of 7-day output with a 5-day hourly forecast at approximately 500 m resolution since June 2010. This high-resolution model is used to capture shallow water effects and nearshore coastal dynamics such as refracting, shoaling, and smaller scale shadowing. Initial boundary conditions for this nested model are obtained from the Hawai’i regional-scale WaveWatch III wave model. From this dataset we extracted for the period ranging from January 2011 to January 2016 the significant wave height (*Hs*) and the mean wave period (*Tm*). We then used linear interpolation to map these 500 m resolution wave parameters on the 30 m Oahu bathymetry map (Figs. S3–2).

Under pure waves (i.e. with no superimposed current), the wave-generated bed shear stress τ_w_ is typically conceived of as a quadratic bottom friction:

14 $${\tau }_{w}=\frac{1}{2}\rho {f}_{w}{U}_{w,b}^{2}$$

where, $\rho$ is water density, ${f}_{w}$ is the wave friction factor, and *U*_*w,b*_ is the maximum over-the-wave-cycle horizontal wave-orbital velocity. Inserting into above equation the linear shallow-water approximation for *U*_*w,b*_, given by:

15 $${U}_{w,b}=({H}_{s}/2)\sqrt{g/h}$$

where, *g* is the acceleration due to gravity and *h* the water depth, yields an expression for **τ**_w_ in terms of the wave height^42^:

16 $${\tau }_{w}=\frac{\rho g{f}_{w}}{8}\frac{{H}_{s}^{2}}{h}$$

Assuming that the wave boundary layer is hydraulically rough turbulent, the wave friction factor, by definition^38^, depends solely on the bed roughness *k*_*b*_ relative to the wave-orbital semi excursion at the bed A_b_. Following Soulsby^21^, we use:

17 $${f}_{w}=1.39{({A}_{b}/{k}_{b})}^{-0.52}$$

where, *A*_*b*_ = *U*_*w,b*_*T*_*m*_ and *k*_*b*_ is evaluated as a grain roughness^42^ given as $2\pi {d}_{50}/12$, where *d*_*50*_ is the median grain size of the bed sediment.

Most waves in the region reach wave base at approximately 20 m depth and convert their wave energy into shear stress across the sea floor, providing a means for mechanical abrasion of both carbonate framework and direct sediment producers. As an example, Figs. S3–2 shows the derived values for horizontal wave-orbital velocity and shear stress obtained from PacIOOS dataset. Based on time series of daily averaged shear stress for the period ranging from January 2011 to January 2016, we infer the modes of transport (wash, suspended and bed loads) based on the Rouse number for the chosen three types of nominal diameter using settling velocities from the equations derived for siliciclastic sand (Eqs. 9 and 6) and the proposed equations (Eqs. 12 and 8) derived for carbonate sands. We then compute for the studied region the annual weekly-averaged percentage of covered area under the influence of each mode of transport (Figs. S3–3).

## Supplementary information

Supplementary Material.

## Data Availability

The dataset employed to optimize Eq. 11 is available upon request from Professor Kwok Fai Cheung.
