# Supplementary material for: Improved drag coefficient and settling velocity for carbonate sands
Source: Sci Rep. 2020 Jun 11;10:9465. doi: 10.1038/s41598-020-65741-3 (PMC7290030; doi:10.1038/s41598-020-65741-3)
Supplement: Supplementary file 1 — Supplementary Material. [file 41598_2020_65741_MOESM1_ESM.pdf]

SUPPLEMENTARY INFORMATION

Improved drag coefficient and settling velocity for carbonate sands

Amin Riazi[1], Ana Vila-Concejo[2*], Tristan Salles[2], Umut Türker[3]

1. Civil Engineering Department, Cyprus International University, Lefkoşa, 99258, North Cyprus, Turkey.
2. Geocoastal Research Group, School of Geosciences, The University of Sydney, Sydney 2006, Australia.
3. Civil Engineering Department, Eastern Mediterranean University, Gazimağusa, 99628, North Cyprus, Turkey.

* Correspondence to ana.vilaconcejo@sydney.edu.au

S1 - Particle shape

Table S1. Comparison of the particles used in the present study and particles used by [1] in terms of particles Corey shape factor.

	Particles Corey Shape factor			
	Minimum	Average	Maximum	Standard Deviation
Smith and Cheung (2003)	0.074	0.556	0.937	0.159
Current study	0.291	0.502	0.758	0.144

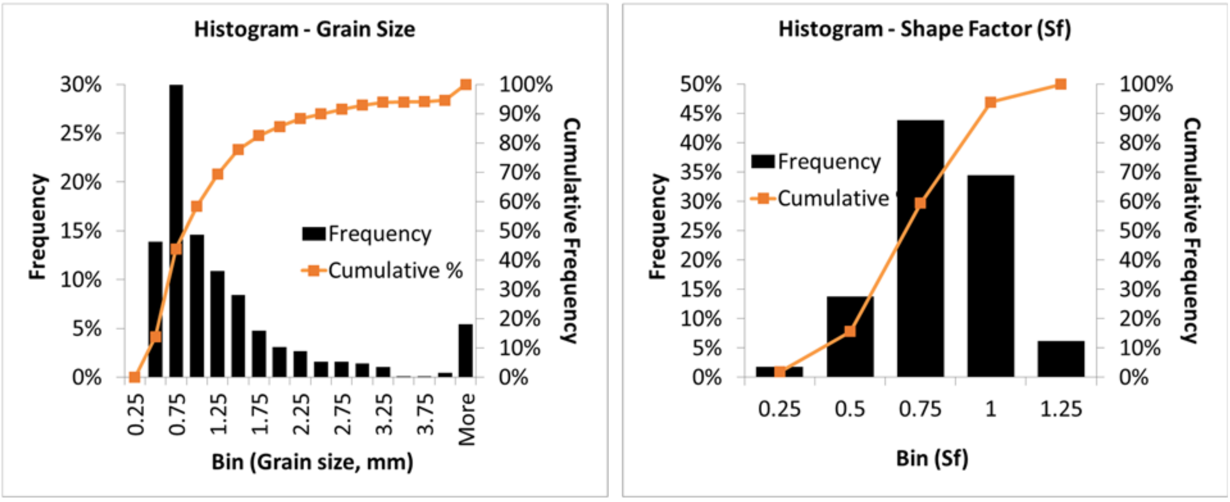

Fig. S1. Histograms of Smith and Cheung's (2003) data showing distribution of grain size (mm) and shape factors. Grain size shows that 92% of the particles have a grain size >0.25 mm and ≤3 mm. The Shape factor shows that 92% of the particles have a shape factor >0.2 and ≤0.8.

S2 - Other settling velocity studies

Alcerreca et al.[2] undertook an empirical study on the settling velocity of carbonate sands. They proposed an empirical settling velocity equation with no functions depending on the shape of the particles.

$$\frac{\omega d_n}{\nu} = \left(\sqrt{22 + 1.13 D_*^2} - 4.67\right)^{1.5} \tag{S1}$$

Table S2. The accuracy of different equations in estimating the settling velocity of carbonate sands over 938 samples.

	No. of data	Riazi and Türker (2019), Eq. 6 and 9	Wu and Wang (2006), Eq. 3	Alcerreca et al. (2013), Eq. S1	Eq. 8 and 12
$0.0<S_f \le 0.2$	17	18.73%	10.95%	53.05%	11.31%
$0.2<S_f \le 0.4$	132	10.59%	11.18%	25.87%	9.29%
$0.4<S_f \le 0.6$	409	9.44%	10.94%	10.02%	9.17%
$0.6<S_f \le 0.8$	322	9.57%	9.34%	11.45%	8.57%
$0.8<S_f \le 1.0$	58	11.30%	10.99%	15.09%	9.54%
Average		9.93%	10.43%	13.83%	9.04%
SD		0.084	0.077	0.136	0.075

S3 – Numerical modelling

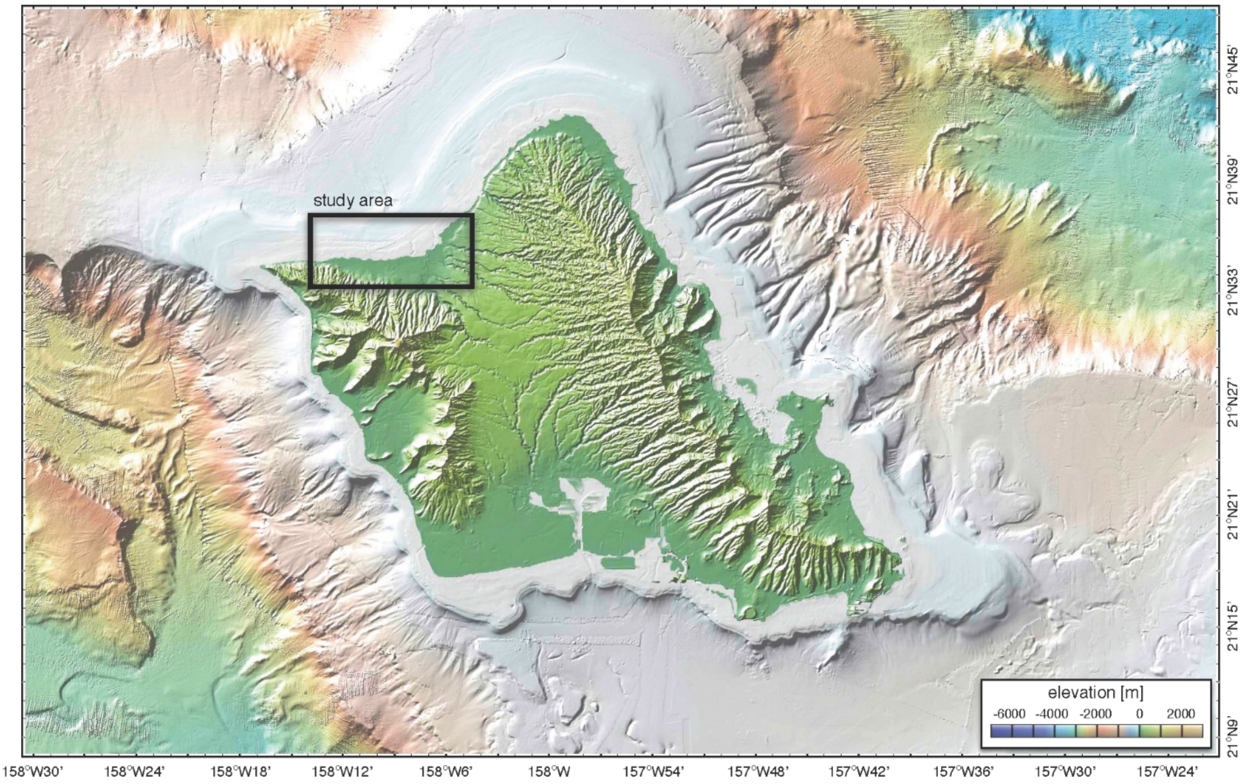

Fig. S3-1. High-resolution map covering Oahu (Hawai'i) at 1-arc-second resolution (approx. 30 m) developed by the NOAA Center for Tsunami Research showing studied area location. Figure made with GeoMapApp (www.geomapapp.org) / CC BY / CC BY [3]

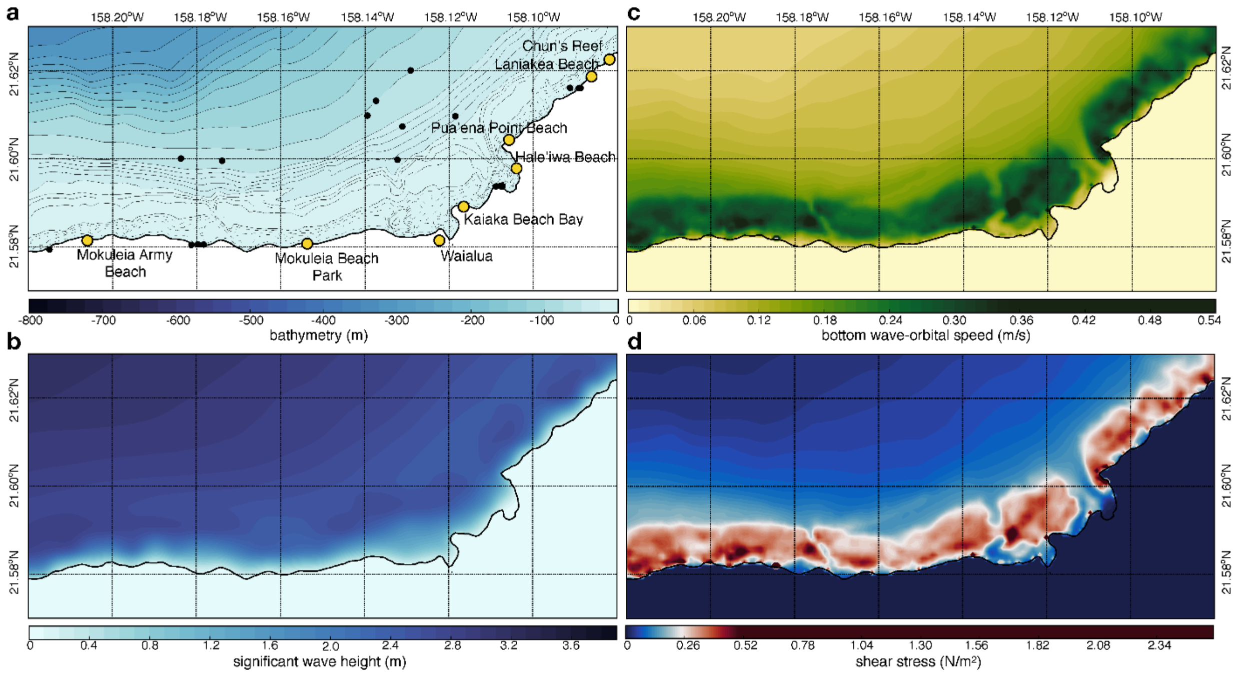

Fig. S3-2. (a) Study area is located on the northwest coast of the island and extent from Mokuleia Army Beach to Chen's Reef from West to East. The bathymetry in the region of interest goes up to 300 m depth on the North but is characterised by a shallow seafloor below 20 m which consists mostly of loose carbonate sands and patchy occurrences of coral and coralline algae growth. Example of wave-induced bottom shear stress calculated from the PacIOOS SWAN wave dataset for the day 02/03/2013 at 3:00 pm. (b) Panel shows the significant wave height (Hs) directly extracted from the dataset. The two right panels (c & d) present the horizontal wave-orbital bottom velocity Uw,b and the wave-generated bed shear stress τw obtained assuming the linear shallow water approximation and pure wave conditions.

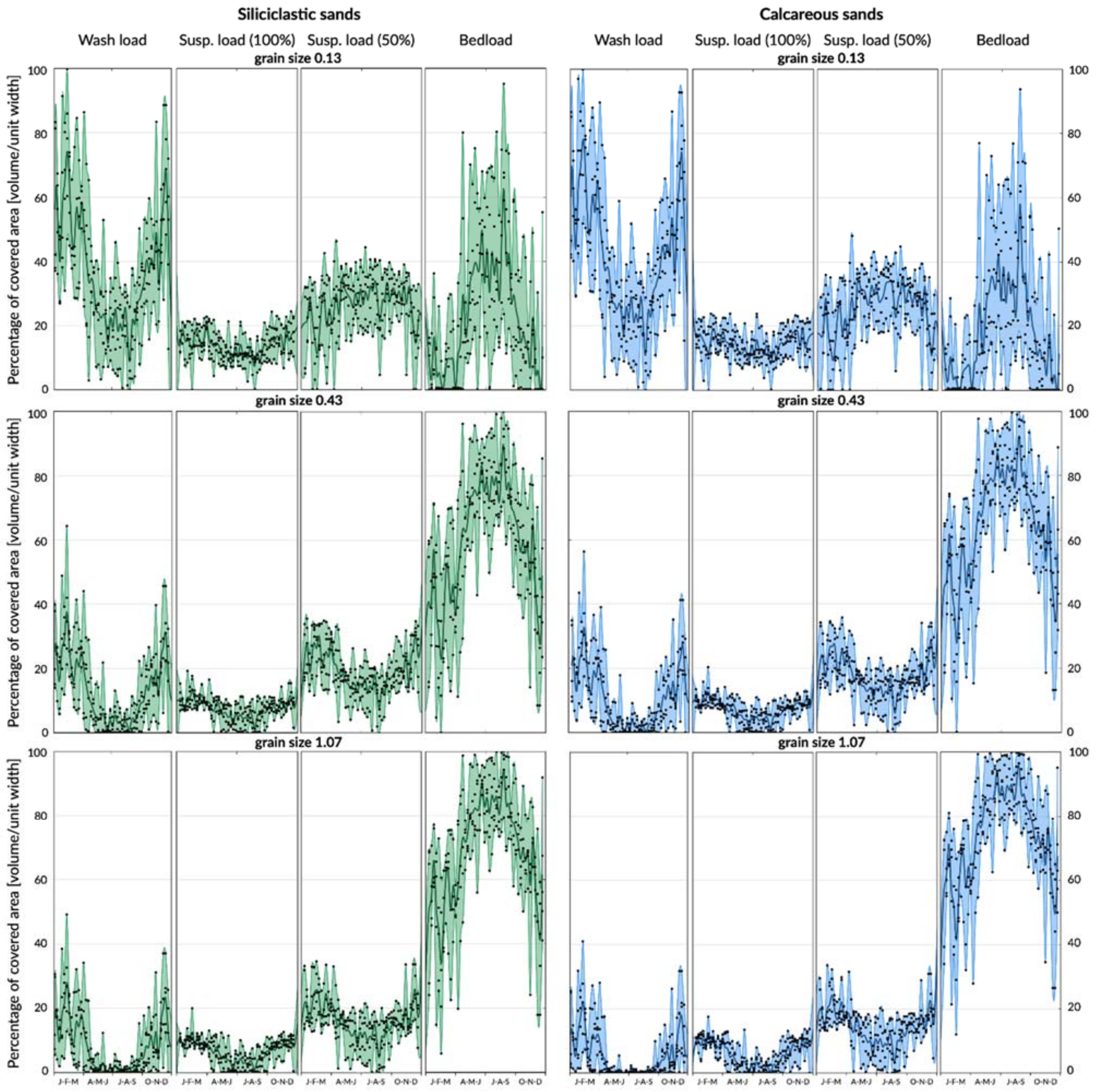

Fig. S3-3. Estimated modes of transport based on settling velocity formulation for siliciclastic (left) and our improved formulation of carbonate (right) sands ranging from January 2011 to January 2016. Each sub-panel presents stacked timeseries for the period of interest of the percentage of area covered by 4 types of mode of transport. For each week, three dots representing the minimum, maximum and mean values are plotted. The strong middle line shows the mean of the weekly estimated percentage of area covered and the shaded coloured zone provides the extent (min/max) of the covered area over time.

LITERATURE CITED

1. Smith, D. a. & Cheung, K. F. Settling Characteristics of Calcareous Sand. *J. Hydraul. Eng.* **129**, 479–483 (2003).

2. Alcerreca, J. C., Silva, R. & Mendoza, E. Simple settling velocity formula for calcareous sand. *J. Hydraul. Res.* **51**, 215–219 (2013).

3. Ryan, W. B. F. *et al.* Global multi-resolution topography synthesis. *Geochemistry, Geophys. Geosystems* **10**, Q03014 (2009).
